# Neuropsychiatric symptoms of the elderly with Alzheimer's disease and the family caregivers' distress**^1^**

**DOI:** 10.1590/1518-8345.0580.2751

**Published:** 2016-08-15

**Authors:** Luana Baldin Storti, Débora Teles Quintino, Natália Michelato Silva, Luciana Kusumota, Sueli Marques

**Affiliations:** 2 RN, Master's Student, Escola de Enfermagem de Ribeirão Preto, Universidade de São Paulo, PAHO/WHO Collaborating Centre for Nursing Research Development, Ribeirão Preto, SP, Brazil.; 3 Undergraduate student in Nursing, Escola de Enfermagem de Ribeirão Preto, Universidade de São Paulo, PAHO/WHO Collaborating Centre for Nursing Research Development, Ribeirão Preto, SP, Brazil.; 4 Psychologist, Master's Student, Escola de Enfermagem de Ribeirão Preto, Universidade de São Paulo, PAHO/WHO Collaborating Centre for Nursing Research Development, Ribeirão Preto, SP, Brazil.; 5 PhD, Professor, Escola de Enfermagem de Ribeirão Preto, Universidade de São Paulo, PAHO/WHO Collaborating Centre for Nursing Research Development, Ribeirão Preto, SP, Brazil.

**Keywords:** Aged, Caregivers, Behavioral Symptoms, Dementia, Geriatric Nursing

## Abstract

**Objective:**

to analyze the relationship between the distress of the family caregiver and the presence of neuropsychiatric symptoms in elderly patients with Alzheimer's disease or mixed dementia.

**Method:**

a descriptive, cross-sectional study conducted in the Geriatric and Dementias Clinic of a general tertiary hospital, with 96 elderly people with Alzheimer's disease or mixed dementia and their family caregivers. Questionnaires to characterize the elderly and caregivers, and the Neuropsychiatric Inventory were used. Descriptive statistics and Pearson correlation test were performed.

**Results:**

68.7% of the elderly were women, average age 80.8 years, 56.2% had Alzheimer's disease and 43.7%, mixed dementia. Among caregivers, 90.6% were women, average age 56, 70.8% took care of parents and 64.6% lived with the elderly. There was a strong (r = 0.82) and significant (p <0.01) correlation between the total score on the Neuropsychiatric Inventory and the total score on the Neuropsychiatric Inventory-Distress and strong (r = 0.80) and significant (p <0 01) correlation between the total score on the Neuropsychiatric Inventory Distress and the number of neuropsychiatric symptoms, i.e., the higher the number, frequency and severity of these symptoms in the elderly, the more intense is the caregiver distress.

**Conclusion:**

the presence of neuropsychiatric symptoms in the elderly was related to increased distress in caregivers.

## Introduction

In the face of process of population aging, there is an increased prevalence of dementia, especially Alzheimer's disease (AD), a neurodegenerative disease characterized by the presence of entanglement and brain plaques, lost connections, inflammation and eventual death of brain cells. Such changes lead to the loss of memory, changes in thinking and other brain functions. The disease progresses gradually and slowly, with cell death, resulting in brain damage. Another type of dementia, which also affects the elderly, is the vascular one resulting from ischemia, hemorrhage, cerebral hypoxia or anoxia. Because of the similarities in symptoms, pathophysiology and risk factors, AD and vascular dementia are not easily distinguished^1^. Considering these factors, many patients manifest the clinical picture of the two dementias, featuring mixed dementia (MD)^2^.

Behavioral and psychological symptoms are common in dementia. This terminology refers to the set of symptoms and signs associated with disorders of perception, the content of thinking, mood or behavior occurring in patients with dementia^3^. Throughout the evolution of AD, neuropsychiatric symptoms appear, such as agitation, depression, hallucinations, delusions and other psychopathological changes, causing suffering to the elderly, morbidities for caregivers and their families and increasing the financial costs of health care^4^.

Regarding the management of behavioral and psychological symptoms of dementia, nursing interventions are needed with regard to guidance to be given to caregivers on how to deal with these symptoms presented by elderly patients with dementia, working out with them specific strategies for each behavioral change^5^. 

Elderly people with dementia often have dependency and disability, and thus need help in their daily activities. People who usually care for the elderly living at home, are members of the family^6^, recognized as family caregivers.

The presence of neuropsychiatric symptoms in the elderly requires from the caregivers skills to deal with them, patience and constant supervision. This situation can lead to physical and emotional stress and hence fatigue that may have a negative influence on various aspects of their life including health^6^.

Considering that the neuropsychiatric symptoms are common in dementia, and they are part of the main reasons for institutionalization, medication use, increased costs of care and burden on the family^7^, it is important to know the relationship between the presence of neuropsychiatric symptoms in AD patients and MD, receiving care in a Clinic of Geriatrics and Dementias, and the distress of the family caregivers. 

Considering this background the question is: what is the relationship between the presence of neuropsychiatric symptoms in AD and MD patients, treated at a Clinic of Geriatrics and Dementias, and the distress of the family caregiver? Thus, from the knowledge of the relationship between the presence of neuropsychiatric symptoms in AD patients and DM and the family caregiver distress, this paper can contribute to the health professionals who work at the clinic, especially nurses, to target care planning with focus on management of behavioral changes of the elderly, in order to minimize the distress of the caregiver. Given the above, the objective of this study was to analyze the relationship between the distress of the family caregiver and the presence of neuropsychiatric symptoms in elderly patients with a diagnosis of AD or DM.

## Methods

Descriptive study with cross-sectional design. Performed at the Clinic of Geriatrics and Dementias of a Tertiary General Hospital, in São Paulo state. The population consisted of elderly patients with a diagnosis of AD or MD treated at the clinic and their respective family caregivers, in the period between November 2013 and April 2014, considering the predefined criteria for inclusion or exclusion. 

Inclusion criteria: a) old age- having 60 years or more, male or female, with a diagnosis of AD or MD in attendance at the clinic and being cared for by a family member; b) caregiver - be the caregiver of an elderly, with AD or MD in attendance in the aforementioned outpatient center and needing home care, male or female and over 18 years. Exclusion criteria: a) institutionalized elderlies; b) caregiver - being a formal caregiver.

For sample selection, convenience sampling was used. From November 2013 to April 2014, 151 elderly patients with a diagnosis of AD or DM were seen at the clinic of the study. Of these, 23 were excluded (19 institutionalized elderly, three accompanied by formal caregivers and one who had no caregiver), eight refusals and 24 losses. Thus, the sample consisted of 96 elderly patients with a diagnosis of AD or MD and 96 family caregivers. 

Data collection was carried out in the period cited above, through interviews, conducted by the researcher and a research assistant, properly trained for the application of the instrument. For the interviews, a data collection instrument was used containing: a) questionnaire put together by the researcher to characterize the elderly and their caregivers, submitted to face validation by experts; b) Neuropsychiatric Inventory (NPI) developed^8^ for the purpose of evaluating the presence, frequency and severity of neuropsychiatric symptoms in patients with dementia. It is composed of 12 domains: delusions, hallucinations, agitation / aggression, dysphoria/depression, anxiety, euphoria/elation, apathy/indifference, disinhibition, irritability, aberrant motor behavior, nocturnal behavior and appetite/eating changes^9^. The scores for the severity of the behavior ranged from 1 to 3, with 1 being slight (behavior is present and causes the patient little discomfort); 2 moderate (more uncomfortable for the patient, but it can be circumvented by the caregiver) and 3 high (behavior is very stressful for the patient and can not be circumvented by the caregiver) and regarding frequency they ranged from 1 to 4, being 1 incidental (less than once per week); 2 common (about once per week); 3 frequent (several times per week, but less than every day) and 4 very frequent (once a day or more). The total score ranges from 0 to 144 points. To assess the emotional and psychological stress of the caregiver, caused by the presence of neuropsychiatric symptoms assessed by the NPI, it has been developed an auxiliary scale^10^, the Neuropsychiatric Inventory Distress (NPI-D). The total score ranges from 0 to 60 points. In Brazil, the NPI and the NPI-D were culturally adapted and validated^9^.

Interviews were conducted on the days and hours of operation of the clinic where the study was conducted, on Fridays, from 13.30 to 18.00. The researcher through hospital records identified elderly people with a diagnosis of AD or MD. After identifying the potential participants, the researcher and research assistant approached the family caregiver and the elderly, identifying themselves, and clarifying the details of the study while inviting them to participate. Then they presented and discussed the Free and Informed Consent Forms (FICF) of the elderly and the caregiver. After clarification and consent of each participant, they were asked to sign the two FICFs and handed a copy of each of them. Considering that the elderlies had medical diagnoses of AD or MD with significant cognitive impairment, only the caregivers answered questions. The average length of the interviews was 45.6 minutes. 

For the processing of the data, a data spreadsheet in Microsoft Excel computer program containing a dictionary (codebook) and two worksheets were prepared in which the data were entered in the form of double entry, to verify the internal consistency of them (double entry validation). After typing and validation, data were exported to the statistical software SAS(r) 9.0, to carry out the distribution of absolute and relative frequencies of all instrument variables and measures of central tendency and dispersion for numerical variables. All statistical analyzes were performed using the statistical software SAS^(r)^ 9.0 and R version 3.0.1.

To verify the correlation between the total score of the NPI and the total score NPI-D and the correlation between the total score NPI-D and the number of neuropsychiatric symptoms, we used the Pearson correlation coefficient, noted as r. The maximum possible value of r is 1, and its minimum value is -1, so -1 ≤ r ≤ 1. In this study, the values ​​adopted for r were: r = -1.0 (perfect negative correlation); r = -0.8 (strong negative correlation); r = -0.5 (negative moderate correlation); r = -0.2 (weak negative correlation); r = 0.0 (No correlation); r = +0.2 (weak positive correlation); r = +0.5 (moderate positive correlation); r = +0.8 (strong positive correlation) and r = +1.0 (perfect positive correlation) ^11^. The significance level for the statistical tests was 5% (p <0.05).

The project was submitted for consideration by the Ethics Committee of the Ribeirão Preto School of Nursing, University of São Paulo under Protocol number 17236613.9.0000.5393, approved in October 2013.

## Results

Regarding the elderly with Alzheimer's disease or MD, their age ranged between 66 and 96 years, with an average of 80,8 years and standard deviation 5,7; the age group most frequent, 56 (58,3%), were those aged 76-85 years. Most, 66 (68,7%) were female. Years of schooling varied between 0 and 15 years with an average of 3.5 and standard deviation 3.7; 54 (56,2%) of 1 to 4 years of study, followed by 16 illiterates (16,7%). As for the type of dementia, 54 (56,2%) of the elderly have a diagnosis of AD and 42 (43,7%), MD. The time with a dementia diagnosis ranged from 1 to 120 months with an average of 32,8 and standard deviation 29,3, the majority, 81 (84,4%), with diagnosis time between 1 and 60 months. 

With regard to family caregivers of elderly patients with AD or MD, their age ranged between 30 and 90 years with an average of 56 and standard deviation 10,6; most of them, 66 (68,7%), were aged between 50 and 69 years. The majority, 87 (90,6%) were female; 63 (65,6%) were married / living with a partner. Years of schooling varied between 0 and 24 years with an average of 9,0 and standard deviation 4,7; 30 (31,3%) of the participants went to school from 9 to 12 years.

As for the care aspects, 68 (70.8%) caregivers surveyed reported taking care of their parents, 62 (64.6%) were reportedly living with the elderly. The total time elapsed since they were taking care of the elderly with Alzheimer's disease or DM ranged from 4 to 456 months, with an average of 78,7 and a standard deviation of 75,2, being the 4-60 months the interval with greater frequency, 55 (57,3%).

**Figure 1:**
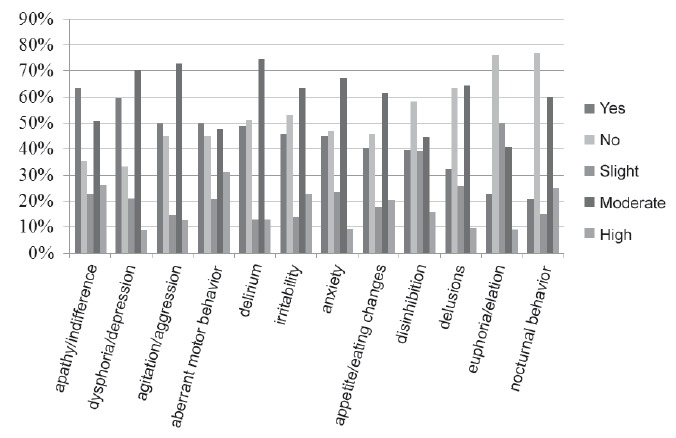
Distribution of neuropsychiatric symptoms of elders with AD or MD by ocurrence and seriousness, Ribeirão Preto, SP, Brazil, 2014

The distribution of occurrence and seriousness of neuropsychiatric symptoms in the elderlies with AD or MD can be seen in Figure 1.

The number of neuropsychiatric symptoms in elderly patients with AD or MD varied between 0 and 11, with an average of 5,0 and standard deviation 2,8. It is noteworthy that, according to family caregivers, 61 (63,5%) of the elderly showed apathy/indifference, 57 (59,4%), dysphoria/depression, 48 (50,0%), agitation/aggression and other 48 (50,0%), aberrant motor behavior. Regarding the severity of the symptoms, there was a predominance of moderate for almost all symptoms except for euphoria/elation 9 (40.9%).

**Figure 2:**
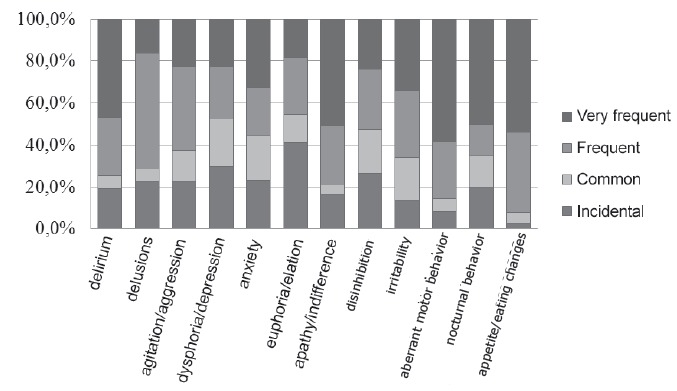
Distribution of neuropsychiatric symptoms of elders with AD or MD by frequency, Ribeirão Preto, SP, Brazil, 2014

The frequency of neuropsychiatric symptoms in AD patients or DM can be seen in Figure 2.

It was observed that, with respect to the frequency of neuropsychiatric symptoms, aberrant motor behavior 28 (58,3%), appetite / eating changes 21 (53,8%), apathy or indifference 31 (50,8%), nocturnal behavior 10 (50,0%) and delirium 22 (46.8%) were mentioned as very common symptoms by family caregivers of elderly patients with AD or DM.

**Figure 3:**
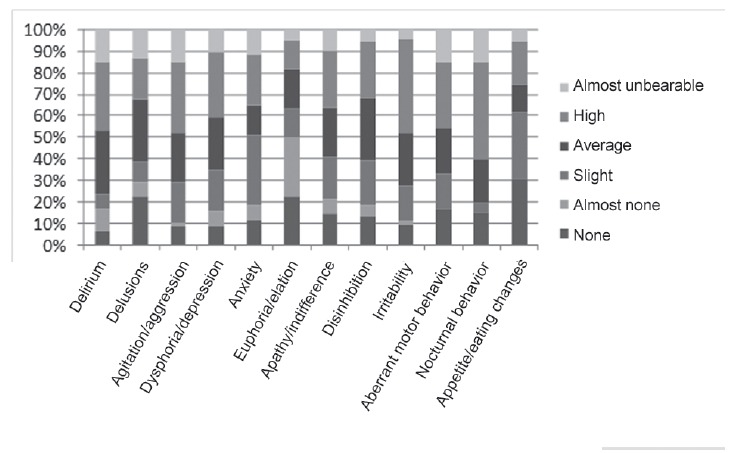
Distribution of neuropsychiatric symptoms of elders with AD or MD by caregivers' distress, Ribeirão Preto, SP, Brazil, 2014

Figure 3 shows the distress of family caregivers, related to neuropsychiatric symptoms presented by the elderly with Alzheimer's disease or MD.

With regard to the caregivers' distress due to the presence of neuropsychiatric symptoms in the elderly, the following number of caregivers reported that those sympoms were a major cause of their distress: nocturnal behavior 9 (45,0%) Irritability 19 (43,2%), agitation 16 (33,3%), delirium 15 (31,9%), and aberrant motor behavior 15 (31,2%).

The total score of the NPI in this study ranged from 0 to 117 points, average 30,0 and standard deviation 23,8. Still, the total score NPI-D ranged between 0 and 48 points, average 14,4 and standard deviation 11,9. 

In this study it was observed that the total score of the NPI and the total score NPI-D are strongly correlated (r = 0,82), i.e., the higher the frequency and severity of neuropsychiatric symptoms presented by the elderly with Alzheimer's disease or MD, the greater the caregiver distress, being statistically significant (p <0,01). It was also evident that the total score NPI-D and the number of neuropsychiatric symptoms presented by elderly patients with AD or MD are strongly correlated (r = 0,80), indicating that the greater the number of neuropsychiatric symptoms presented by the elderly with AD or MD, the greater the caregiver distress, being statistically significant (p <0,01).

## Discussion

Neuropsychiatric symptoms are common in dementia and are a source of burden on the family in elderly care^7^. And the higher the number, frequency and severity of these symptoms in the elderlies, the larger is the caregiver distress.

The analysis of the relationship between the family caregiver distress and the presence of neuropsychiatric symptoms in elderly patients with dementia is relevant given the current epidemiological profile. Worldwide, the prevalence rates of dementia, especially AD, are increasing rapidly, being higher in the Americas compared to less developed regions rates, as in Africa^1^.

In this study, the strong and significant correlation between the total score NPI-D and the number of neuropsychiatric symptoms, shows that the number of neuropsychiatric symptoms interferes with the caregiver distress. It is understood that the caregiver who cares for an senior person that has more than one neuropsychiatric symptom present increased distress, as this elderly may require more care.

Throughout the evolution of dementia, seniors may have different clinical manifestations such as changes in emotions, mood, perception, thinking, motor activity and personality. These changes result in a high level of distress for the elderlies and their caregivers, as well as increased use of health services^12^.

Regarding the presence of neuropsychiatric symptoms presented by the elderly with Alzheimer's disease or MD, apathy/indifference was the symptom mainly referred by their family caregivers, finding similar to other studies^9^ ^,^ ^13^. Apathy is characterized by indifference and inactivity, which can lead to the distress of the caregiver, because of the sense of frustration on the limitations that the elderly with apathy can present^13^. 

The presence of different neuropsychiatric symptoms in elderly people carry different distress patterns in caregivers^13^. The aberrant motor behavior and nocturnal behavior, for example, are highly stressful because they require higher physical demands of the caregiver. Delusions of theft and identification cause greater psychological stress for the caregiver due to the disability of the elderlies in identifying them^13^.

Importantly, the presence of neuropsychiatric symptoms in the elderly is related to the higher degree of cognitive impairment and advancing dementia, reducing the quality of life of the elderly and raising the stress of the caregiver^14^. 

The caregivers indicated that the aberrant motor behavior as a very common symptom. This finding corroborates another study^15^. This symptom is characterized by increased psychomotor activity, iterative and frequently without purpose^12^. It is noteworthy that, in addition to the caregiver having to live daily with the elder, performing care activities, the increased frequency of neuropsychiatric symptoms in the elderly requires constant supervision, increasing the physical and emotional stress of the caregiver.

Regarding the severity of the symptoms, there was a predominance of the moderate group, that is, the symptoms cause more discomfort to the patient, but can be overcome by the caregiver. This data shows that for the caregivers in this study, neuropsychiatric symptoms can interfere with the welfare of the elderly. This data also reveals that the studied family caregivers are able to deal with seniors who have neuropsychiatric symptoms. 

As for the caregiver distress related to the presence of neuropsychiatric symptoms in the elderly, the nocturnal behavior was appointed as a high-wearing symptom for the caregivers. Different from other studies^13^ ^,^ ^15^ ^-^ ^16^ that revealed delirium, apathy and agitation respectively as the main distressing symptoms for caregivers.

The strong and significant correlation between the total score of the NPI and the total score NPI-D is in concordance with other studies^13^ ^,^ ^16^. As already mentioned, the frequency of neuropsychiatric symptoms in the elderly may interfere with the caregiver distress, showing that the increased frequency of neuropsychiatric symptoms will require constant supervision to the elderly, which in turn may increase the physical and emotional stress of the caregiver.

Demented elderly often have decline in their cognitive functions and behavioral symptoms increase over many years. For caregivers, cognitive decline in the elderly can lead to increased stress, frustration, anxiety, depression and health problems. Thus, providing support to caregivers to cope with stress and emotional care challenges can provide benefits to caregivers^17^.

Some characteristics of the elderly or caregivers relate to the context of care, and in this study we became aware of the presence of elderly caregivers taking care of elderly patients with AD or MD. When there is an elder caring for other elder, the practice of care may become more troublesome, since the elderly caregivers also have their own limitations of the natural aging process, which can compromise the quality of care and caregivers' wellbeing.

According to the literature^16^ ^,^ ^18^, family caregivers are mostly women and middle-aged, fact corroborated by this study. Historically, care is a female task, rooted in our culture, pertaining to women the care of their children, the elderly and sick, as well as household chores. Despite the social and family changes in society, such as the insertion of the women in the labor market, they still stand out as the main responsible for the care of their families^19^.

When care is shared between the spouses, the marital status can be a way of support to caregivers, but when this does not occur, the practice of care can be a factor that interferes with the health of the caregivers, since they do not have available time to care for themselves. In addition to exercising the task of elderly care, caregivers perform other tasks such as caring for children, household tasks, preparation of meals, among others, which can cause overload^19^.

 Note that the caregivers of this study showed a high level of education. This may improve the care activity for the elderly, by facilitating access to information, health education and understanding of the disease by the caregiver^18^.

With regard to the degree of kinship with the elderly, the majority of respondents reported caring for their parents. This can be explained by the fact that exercising the role of caregivers is associated to compliance with social standards on filial duties, as well as the emotional bond between the elderly and the caregiver^18^.

Given the above, the role of health professionals is necessary regarding guidance to caregivers for early recognition and management of neuropsychiatric symptoms in the elderly, which may favor the treatment of such symptoms in order to control and mitigate them and thus contributing to the caregivers and the elderlies'well-being. 

Therefore, early recognition of neuropsychiatric symptoms by the family and health professionals, as well as the immediate implementation of different treatment strategies may facilitate performing a more appropriate care and improve the quality of life for seniors and their caregivers^(13 )^. 

## Conclusion

In this study, we observed a strong correlation between the frequency and severity of neuropsychiatric symptoms and caregiver distress, as well as between the number of these symptoms and caregiver distress. These data reinforce the notion that the presence of neuropsychiatric symptoms in the elderly was related to increased distress on the caregiver.

Regarding the limitations of this study, it is emphasized that the results obtained reflect a local reality, so generalizations should be viewed with caution, in order to avoid misunderstandings.

It is believed that the results of this research show the importance of knowing the relationship between the distress of the family caregiver and the presence of neuropsychiatric symptoms in elderly patients with a diagnosis of AD or MD, in order to obtain the necessary inputs for planning of nursing care for the elderlies and their family caregivers, focusing on the management of behavioral changes, in order to minimize the caregiver distress, allowing to improve the quality of care for the elderly at home and living conditions of both caregivers and elderlies.
